# A Reporter Assay in Lamprey Embryos Reveals Both Functional Conservation and Elaboration of Vertebrate Enhancers

**DOI:** 10.1371/journal.pone.0085492

**Published:** 2014-01-09

**Authors:** Hugo J. Parker, Tatjana Sauka-Spengler, Marianne Bronner, Greg Elgar

**Affiliations:** 1 Division of Systems Biology, Medical Research Council National Institute for Medical Research, London, United Kingdom; 2 Division of Biology, California Institute of Technology, Pasadena, California, United States of America; CNRS, France

## Abstract

The sea lamprey is an important model organism for investigating the evolutionary origins of vertebrates. As more vertebrate genome sequences are obtained, evolutionary developmental biologists are becoming increasingly able to identify putative gene regulatory elements across the breadth of the vertebrate taxa. The identification of these regions makes it possible to address how changes at the genomic level have led to changes in developmental gene regulatory networks and ultimately to the evolution of morphological diversity. Comparative genomics approaches using sea lamprey have already predicted a number of such regulatory elements in the lamprey genome. Functional characterisation of these sequences and other similar elements requires efficient reporter assays in lamprey. In this report, we describe the development of a transient transgenesis method for lamprey embryos. Focusing on conserved non-coding elements (CNEs), we use this method to investigate their functional conservation across the vertebrate subphylum. We find instances of both functional conservation and lineage-specific functional evolution of CNEs across vertebrates, emphasising the utility of functionally testing homologous CNEs in their host species.

## Introduction

The sea lamprey, *Petromyzon marinus*, is a member of the jawless fish lineage (agnathans), the only extant vertebrate sister group to the jawed vertebrates (gnathostomes) [1]. As such, it provides a unique window into early vertebrate history, enabling inference of the ancestral states and evolutionary origins of vertebrate characters. For example, investigations into lamprey genetics and embryogenesis have shed light on the evolution of the jaw [2], [3], paired fins [4], neural crest [5], [6], pharynx [7], immune system [8], sympathetic nervous system [9], forebrain [10], [11], and hindbrain [12]. Whilst the restricted summer breeding season presents some practical difficulties for studying lamprey embryology, the availability of copious embryos during this period has enabled the establishment of a suite of developmental biology techniques for lamprey, including *in-situ* hybridisation, morpholino-mediated gene knockdown and cell labelling [5], [7], [13] [14]. Furthermore, the recent genome assembly has significantly enhanced its utility as a model organism [15].

The emergence and elaboration of *cis-*regulatory elements in early vertebrates are likely to have contributed significantly to the evolution of the vertebrate body plan. Whilst embryonic gene expression patterns can be informative as to the developmental changes underlying morphological evolution, the changes at the *cis-*regulatory level that are responsible for divergent gene expression have received less attention. There are a handful of studies where early vertebrate *cis-*regulatory evolution has been addressed through testing amphioxus or lamprey genomic sequences for enhancer activity in gnathostomes [16]–[18]. These elements provide evidence for ancient conserved mechanisms of gene regulation across chordates. However, in order to gain a more complete picture of the contribution of changes in both *cis-* and *trans*- regulatory state during vertebrate evolution, it is important also to test such elements in their host species [19]. As increasing numbers of lamprey *cis-*regulatory elements are uncovered [20], [21], an efficient reporter assay methodology for functionally testing them in lamprey is sorely needed.

Shared conserved non-coding elements (CNEs), have been identified through comparisons between lamprey and gnathostome genomic sequences, representing a collection of putative *cis-*regulatory elements that were present in the last common ancestor of all vertebrates [20], [21], [22]. As the vast majority of these elements are not identifiable in invertebrate genomes, they are a vertebrate-specific character [23]. Many CNEs have been shown to function as developmental enhancers by reporter assay within their host species (e.g. [23], [24]), yet the degree to which the regulatory functions of CNEs are conserved between species has been investigated less frequently (e.g. [25]). Recent findings from zebrafish and mouse assays suggest that the regulatory functions of orthologous CNEs in their respective species are frequently well conserved, but on occasion can differ considerably [19], [26], which echoes findings from testing paralogous pairs of CNEs in a zebrafish assay [27], [28]. For the instances where homologous CNEs show functional divergence, these elements could provide a route through which divergence in gene expression patterns can be directly linked to specific sequence changes within *cis-*regulatory elements (e.g. [29]). Thus, lamprey-gnathostome CNEs could be informative as to the functional elaboration of homologous *cis*-regulatory elements across the vertebrate sub-phylum.

Kusukabi *et al*. [30] conducted the first application of transgenesis in an agnathan, using a close relative of the sea lamprey – the Japanese lamprey, *Lampetra japonica*. In their study, circular plasmid constructs containing the GFP coding sequence downstream of either a viral promoter or upstream regulatory regions of medaka actin genes were injected into fertilised eggs before the first cleavage, resulting in mosaic GFP expression. Interestingly, muscle-specific actin promoters from medaka were able to drive GFP expression in the developing muscle of lamprey embryos. Whilst the long lamprey life-cycle impinges upon the likelihood of generating transgenic lines, this study illustrated the feasibility of assaying CNEs for enhancer activity in lamprey.

In a study focusing on putative Hox-regulated enhancers conserved between jawed vertebrates and lamprey, we presented reporter expression patterns driven by two lamprey elements in lamprey embryos, verifying that ancient CNEs have broad functional conservation across vertebrates [31]. In this report, we expand upon that by describing the development of the I-SceI meganuclease-mediated transient transgenesis approach for lamprey embryos. We add versatility to the assay by identifying multiple minimal promoters that are functional in lamprey. As an example of its application, we perform reciprocal cross-species comparisons of CNE enhancer activity, finding evidence for both functional conservation and divergence across vertebrate taxa.

## Materials and Methods

### Zebrafish transgenesis

CNEs were amplified from genomic DNA by PCR (primer sequences are given in Figures S1 and S2) and transferred into the pGW_cfosEGFP vector by Gateway recombination via the pCR8/GW/TOPO TA entry vector (Invitrogen). Transgenesis was carried out according to the protocol of Fisher *et al*. [32] using the wild-type QMWT zebrafish strain.

### Lamprey transgenesis

For linearised plasmid injection, the pm3285_cfos_EGFP plasmid, consisting of the lamprey homolog of CNE 3285 cloned into the pGW_cfosEGFP vector, was linearised with KpnI (NEB), purified with a Qiagen PCR purification kit and eluted in distilled water. Lamprey embryos were obtained as described previously [6] and injected with approximately 2–3 nl of linearised plasmid at a concentration of 100 ngμl^−1^ during the first cell division. Circular plasmid injection was performed at a concentration of 50 ngµl^−1^. I-SceI meganuclease-mediated transgenesis was based on the protocol of Ogino *et al*. [33]. Putative enhancers were cloned into the cfos_I-SceI_EGFP lamprey reporter plasmid, engineered for this study, upstream of the mouse *c-Fos* promoter. 20 μl restriction digests containing 15 units I-SceI enzyme (NEB), 2 μl 10X I-SceI buffer, 0.2 μl 100X BSA and 400 ng of reporter plasmid (final concentration 20 ngμl^−1^), were incubated at 37°C for 40 minutes prior to being immediately injected into lamprey embryos during their first cell division (drop volume approximately 2–3 nl).

### Lamprey in-situ hybridisation

performed according to published protocols [14] using the *meis1/2 a* and *b* probes [6].

## Results and Discussion

### Identification of a minimal promoter, c-Fos, that functions in lamprey

We sought to develop a functional assay to test the ability of lamprey homologs of two CNEs, pm3285 and pm3299, associated with the *meis2* locus, to drive reporter expression in lamprey embryos. These CNEs are highly conserved between jawed vertebrates and lamprey [20] (Multiple sequence alignments are provided in Figures S1 and S2) and drive specific patterns of GFP expression in a zebrafish reporter assay. pm3285 drives expression in the cranial ganglia, hindbrain and spinal cord, and pm3299 in the anterior hindbrain [31]. These patterns are consistent with the endogenous expression of *meis2* in zebrafish, which is broadly expressed in the central nervous system, particularly in the hindbrain [34], [35].

**Figure 1:**
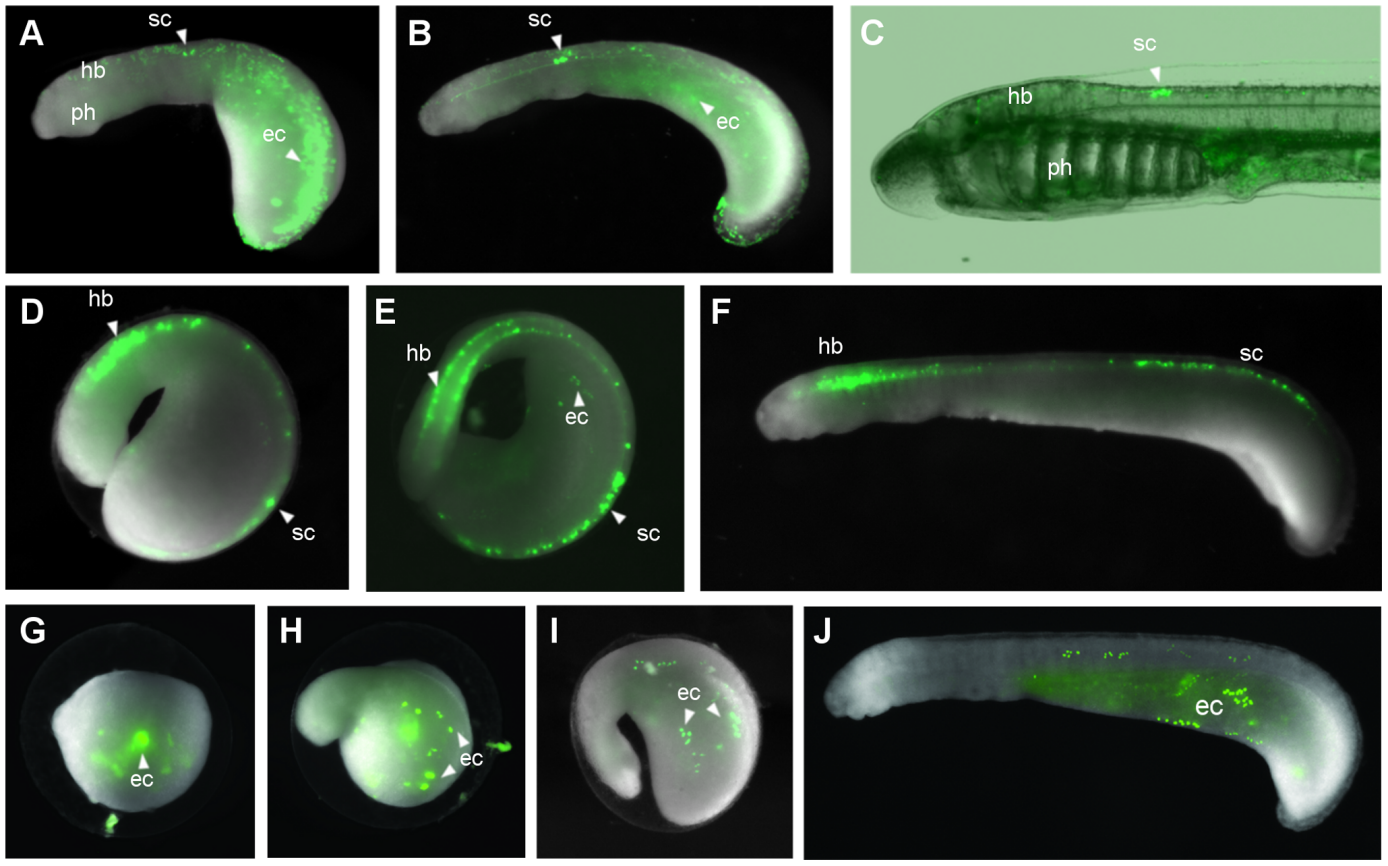
I-sceI meganuclease-mediated transgenesis in lamprey embryos. (**A–C**) Transient transgenic lamprey generated through injection of the linearised pm3285_cfos_EGFP plasmid showing mosaic GFP expression in the ectoderm and in neurons of the spinal cord at stage 23 (**A**), stage 25 (**B**) and at the ammocoete larval stage (**C**). (**D–F**) Lamprey embryo with GFP expression in the hindbrain and spinal cord at stage 23 (**D**), stage 24 (**E**) and stage 25 (**F**), obtained using the meganuclease assay with the pm3285 enhancer. (**G–J**) ‘Background’ GFP expression in the ectoderm of lamprey embryos at stage 19 (**G**), 21 (**H**), 23 (**I**) and 25 (**J**), driven by the cfos_IsceI_EFGP plasmid with no enhancer using the meganuclease method (embryos shown are different individuals). Abbreviations: ec, ectoderm; hb, hindbrain; ph, pharynx; sc, spinal cord.

In order to test these elements for enhancer activity in lamprey embryos, we sought a minimal promoter that can function in lamprey. We tested the suitability of the mouse *c-Fos* minimal promoter, which we used in the zebrafish assay, by injecting the pm3285_cfos_EGFP plasmid into lamprey embryos. The plasmid was linearised to increase the chance of genomic integration. Injection of this construct during the first cleavage resulted in a high death rate immediately post-injection, as well as during gastrulation, such that the frequency of injected embryos surviving through gastrulation was very low (Table 1) and they were often deformed. Injecting the plasmid at lower concentration (50 ngµl^−1^) produced surviving embryos but none were expressing GFP. In a proportion of the survivors from the 100 ngµl^−1^ injections, GFP was expressed in a mosaic manner in the cranial ganglia and neurons of the spinal cord (Table 1), in agreement with the reporter expression driven by the same construct using the Tol2 assay in zebrafish. One of these embryos is shown in Figure 1 A–C, exhibiting GFP expression in neurons of the spinal cord, as well as broad reporter expression in the ectoderm from an early stage. Interestingly, the neuronal expression persisted up to the ammocoete stage in this embryo (Figure 1C). The successful generation of embryos exhibiting GFP expression mediated by this construct suggested that the mouse *c-Fos* minimal promoter is functional in lamprey embryos.

**Table 1:** A comparison of lamprey transgenesis methods.

Method	Element	Promoter	Plasmid conc. /ngul^−1^	Embryos injected	Survivors (% of injected)	Ectodermal expression (% of survivors)	Neuronal expression (% of survivors)
Linearised plasmid	pm3285	cfos	100	500	18 (4%)	10 (56%)	5 (28%)
Linearised plasmid	pm3285 (repeat)	cfos	100	500	17 (3%)	11 (65%)	2 (12%)
Meganuclease	no enhancer	cfos	20	350	232 (66%)	203 (88%)	0
Meganuclease	pm3285	cfos	20	600	220 (37%)	not counted	35 (16%)
Meganuclease	pm3285 (repeat)	cfos	20	550	422 (77%)	166 (39%)	83 (20%)
Circular plasmid	pm3285	cfos	50	550	120 (22%)	0	0
Circular plasmid	pm3285 (repeat)	cfos	50	500	72 (14%)	0	0
Meganuclease (same embryo batch as for circular vector above)	pm3285	cfos	20	500	139 (28%)	not counted	22(16%)
Meganuclease	pm3299	cfos	20	700	302 (43%)	not counted	56 (19%)
Meganuclease	dr3285	cfos	20	600	217 (36%)	not counted	29 (13%)
Meganuclease	dr3299	cfos	20	650	467 (72%)	not counted	49 (10%)
Meganuclease	pm3299	hsp70	20	600	124 (21%)	not counted	44 (35%)

Results are shown for linearised plasmid injection and meganuclease-mediated transgenesis. Ectodermal background expression was not counted for the injections of pm3285 and pm3299 with the meganuclease method, but the proportion of embryos with this background expression was in keeping with that found for the cfos_I-SceI_EGFP construct.

### The effect of I-SceI meganuclease-mediated transgenesis on mosaicism

In an effort to reduce mosaicism and increase embryo survival rate, we applied the I-SceI meganuclease-mediated transgenesis method to lamprey embryos. We reasoned that by increasing the probability of early genomic integration of the injected construct, the amount of DNA injected could also be lowered, lessening the toxic effect of exogenous DNA whilst decreasing mosaicism. This method utilises the rare-cutting I-SceI meganuclease and requires a construct in which the DNA fragment to be integrated is flanked by I-SceI recognition sites. The construct is digested with the meganuclease enzyme *in-vitro* and the reaction mix is then injected immediately into fertilised eggs. Whilst the integration mechanism is unclear, it is unlikely that the enzyme cuts genomic DNA, as its 18 bp recognition site is predicted to occur once in every 7×10^10^ bp of genomic sequence [36] (compared to the lamprey genome size of 2.3×10^9^ bp). Rather, the continued association of the enzyme with the digested construct may prevent its degradation or concatamerisation, thus increasing the probability of genomic integration [36].

When pm3285 was tested using the meganuclease assay, the embryo survival was considerably higher than with linearised plasmid (37% survival post-gastrulation compared to 3.6%) and 35 embryos showed expression in the hindbrain or spinal cord, with 10 of these 35 also expressing GFP in the cranial ganglia (Table 1). Repeat injections confirmed that the increased embryo survival achieved through the meganuclease approach is reproducible (Table 1). The pattern of GFP expression obtained using the meganuclease method is in the same domains as that obtained by the injection of the pm3285_cfos linearised plasmid (Figure 1A–C compared to D–F). The earliest neural expression is seen at stage 21 in a low number of cells, becoming more expansive at later stages, whilst most embryos also display mosaic ectodermal expression.

The transient transgenic embryos obtained through the meganuclease approach display a range of mosaicism with respect to GFP expression in neurons. To compare mosaicism between the two transgenesis approaches, we categorised transient transgenics into two groups depending on whether they had more than or less than 50 GFP-positive neurons at stage 24. For the linearised plasmid injection, 0/5 embryos with neuronal GFP expression exhibited this expression in more than 50 neurons. In contrast, for the meganuclease approach, 9/35 embryos with neuronal expression had more than 50 neurons expressing GFP (examples of transient transgenic embryos obtained from each method are shown in Figure S3). This suggests that the meganuclease approach yields transient transgenic embryos with decreased mosaicism compared to linearised plasmid injection. However, due to the low number of survivors obtained through linearised plasmid injection, it is unclear whether its apparently high mosaicism is a general trend. Nevertheless, in this instance, the meganuclease approach was beneficial in generating an appreciable number of GFP-expressing embryos that showed lower mosaicism than those obtained through linearised plasmid injection. We were unable to generate any GFP-expressing lamprey embryos through injecting the pm3285_cfos_I-SceI_EGFP construct as a circular plasmid without the I-sceI meganuclease enzyme, an approach that had been used by Kusukabi *et al*. (2003) [30] with a different construct (Table 1). Thus, for our construct, the meganuclease transgenesis approach represents an improvement over circular plasmid injection in terms of the frequency of obtaining transient transgenic embryos, and an improvement over linearised plasmid injection in terms of the balance of reporter expression and embryo survival. Importantly, the promoter control – the cfos_I-SceI_EGFP construct with no enhancer – when injected using the meganuclease method, resulted in a large proportion (87.5%) of the survivors showing mosaic GFP expression in the ectoderm and yolk, but not in any other domains (Table 1, Figure 1G–I). As this expression is driven by the construct in the absence of an enhancer, we consider it to be ‘background’ expression.

### Effects of CNE sequence divergence on transgene expression

**Figure 2:**
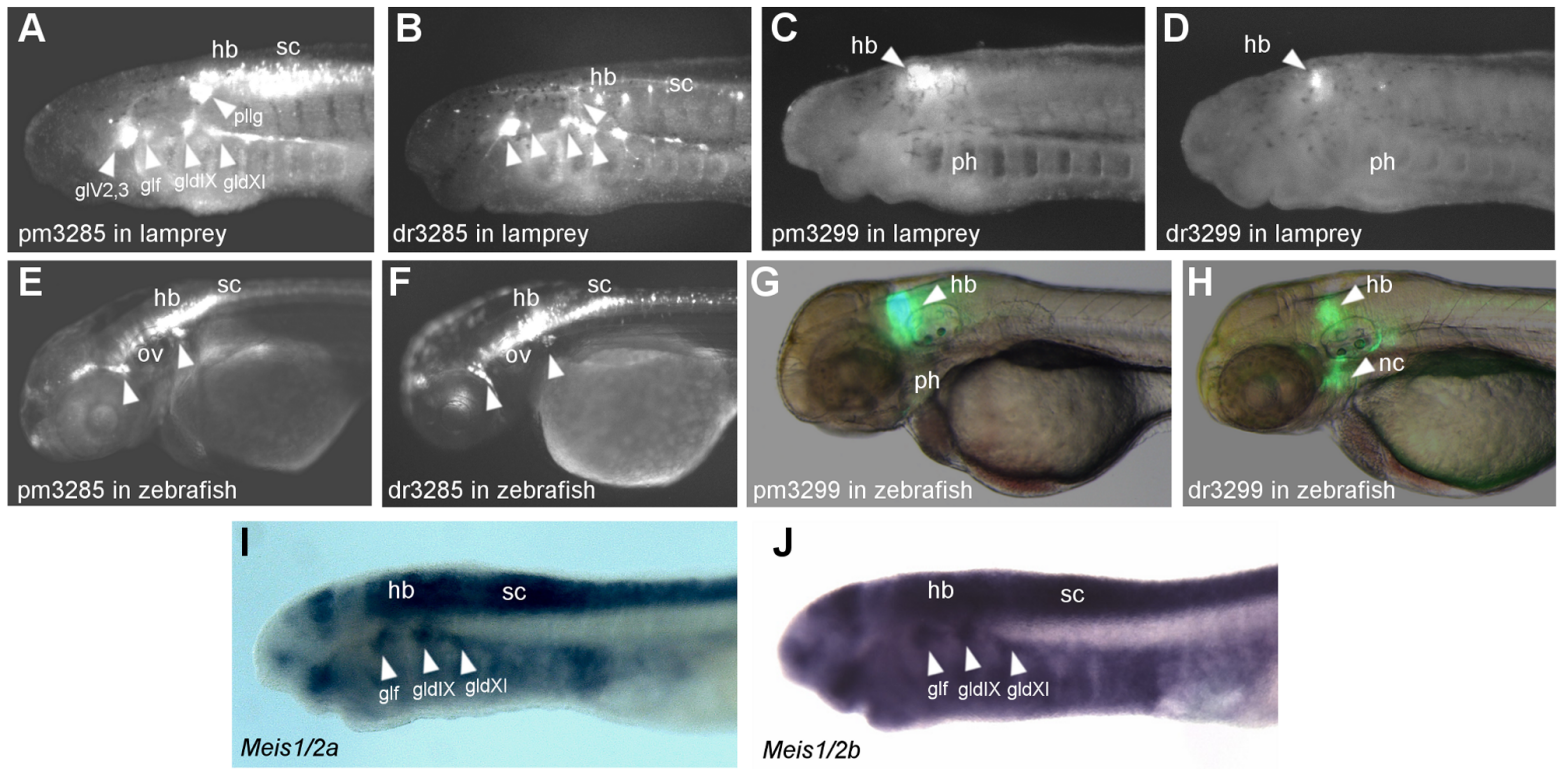
Reporter expression driven by CNEs 3285 and 3299 in lamprey and zebrafish embryos. (**A–B**) GFP fluorescence in stage 26 transient transgenic lamprey embryos, generated by meganuclease-mediated transgenesis with the pm3285 (**A**) and dr3285 enhancers (**B**). GFP expression is seen in the cranial ganglia (arrowheads), hindbrain and spinal cord. (**C–D**) GFP fluorescence in the rostral hindbrain in stage 26 transient transgenic lamprey embryos generated by meganuclease-mediated transgenesis with the pm3299 (**C**) and dr3299 (**D**) enhancers. GFP expression driven by pm3285 (**E**) and dr3285 (**F**) in 54hpf F1 transgenic zebrafish embryos, created by Tol2 transgenesis. Expression in the cranial ganglia is seen for the elements from both zebrafish and lamprey (arrowheads). Expression is also seen in primary neurons of the hindbrain and spinal cord. (**G–H**) 54hpf F1 transgenic zebrafish embryos showing GFP expression driven by the pm3299 (**G**) and dr3299 enhancers (**H**). GFP expression in the hindbrain is driven by both elements, whilst only the zebrafish element up-regulates GFP in the neural crest. (**I–J**) Expression of *Meis1/2a* (**I**) and *b* (**J**) genes in stage 25 lamprey embryos, revealed by *in-situ* hybridisation. Arrowheads highlight expression in cranial ganglia. Abbreviations: glV2,3, trigeminal ganglion; gldIX, epibranchial ganglion of the glossopharyngeal nerve; gldX1, epibranchial ganglion of the vagus nerve; glf, facial ganglion; hb, hindbrain; nc, neural crest; ov, otic vesicle; ph, pharynx; pllg, posterior lateral line ganglion; sc, spinal cord.

To address whether CNE enhancer function has diverged between vertebrate lineages, we have focused upon the reporter expression patterns driven by the zebrafish (dr) and lamprey (pm) CNEs when tested in zebrafish and lamprey embryos. As the same CNE sequences were used in each assay, the expression patterns driven by them are directly comparable [28]. For each CNE, at least two independent transgenic lines were generated and their expression domains were compared. We observed GFP expression from pm3299 in 3 lines, dr3299 in 3 lines, pm3285 in 2 lines and dr3285 in 2 lines. The tissue specific domains of reporter expression that we highlight were conserved between lines for each enhancer. Expression in the cranial ganglia driven by pm3285 and dr3285 (Figure 2A,B), is in agreement with the reporter expression seen in transgenic zebrafish lines generated with these elements, in which GFP is seen in clusters of cranial ganglia both anterior and posterior to the otic vesicle (Figure 2E,F). Two lamprey genes showing homology to jawed vertebrate *meis* genes have been identified and named *pmMeis1/2a* and *b* [6]. The expression patterns of these two genes at this developmental stage are very similar to each other, with both showing clear cranial ganglia expression (Figure 2I,J). However, the expression of these genes does not entirely overlap with the GFP expression driven by pm3285, which extends to more anterior cranial ganglia. Both pm3285 and dr3285 also drive expression in neurons of the hindbrain and spinal cord in lamprey embryos (Figure 2A,B), expression domains that are also seen for these elements in zebrafish (Figure 2E,F). The reporter expression patterns driven by these elements when tested in zebrafish and lamprey embryos suggests that, with respect to these elements, *cis-* and *trans*-regulatory state are broadly conserved between lamprey and gnathostomes.

The hindbrain expression driven by pm3299 in lamprey has an anterior limit consistent with the expression pattern of the two lamprey *meis* genes (Figure 2C,I,J). In transgenic zebrafish lines, the expression driven by dr3299 differs from that of its lamprey homolog in two regards: firstly, dr3299 drives expression in the neural crest cells settling in the hyoid arch, whilst pm3299 does not (Figure 2G,H); secondly it is restricted to a smaller domain of the hindbrain, whilst pm3299 drives broader hindbrain expression (Figure 2G,H). These patterns led us to speculate that this enhancer may have been elaborated in gnathostomes relative to lamprey, such that it gained a new expression domain in the neural crest and its expression in the hindbrain became more restricted. No observable neural crest expression is driven by pm3299 in lamprey embryos (Figure 2C), and the broad pattern of hindbrain expression (Figure 2C) is in line with the reporter expression driven by pm3299 in zebrafish (Figure 2G). The zebrafish element, dr3299, when tested in lamprey, drives reporter expression in the hindbrain but no reporter expression is seen in the pharyngeal neural crest (Figure 2D). This suggests that the neural crest expression that is driven by the zebrafish enhancer when tested in zebrafish is a consequence of differences in both *cis* and *trans* between zebrafish and lamprey.

### Effects of different promoters on transgene expression

**Figure 3:**
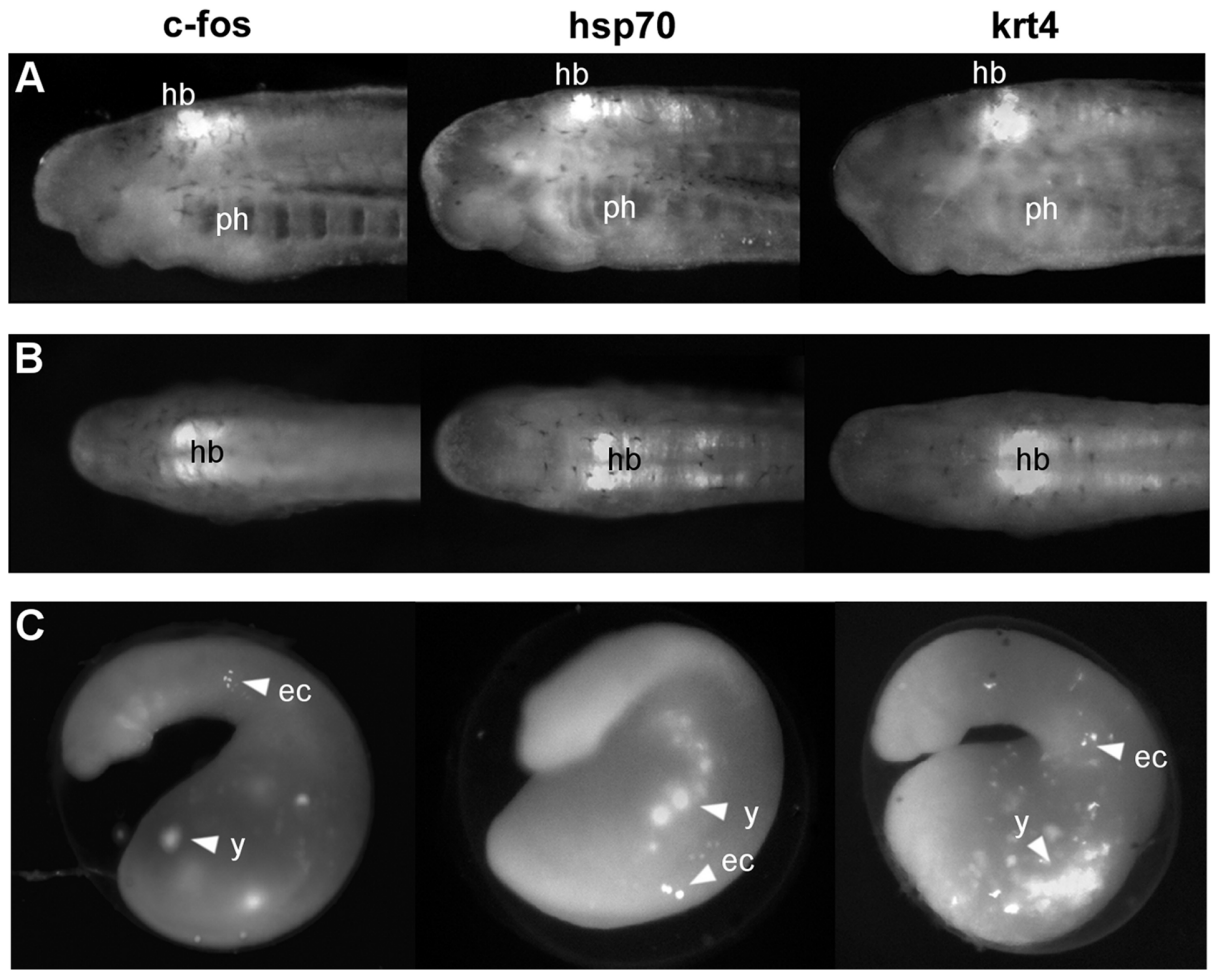
Identifying minimal promoters that are functional in lamprey. (**A–B**) Lateral (**A**) and dorsal (**B**) views of stage 26 transient transgenic lamprey embryos, showing reporter expression in the anterior hindbrain driven by different minimal promoters in conjunction with the pm3299 enhancer using the I-SceI meganuclease approach. (**C**) Lateral views of stage 23 lamprey embryos injected with the same constructs as those in **A** and **B**, showing early expression in ectoderm and yolk, similar to the background expression driven by the control cfos_I-SceI_EGFP construct (see Figure 1I). The identity of the minimal promoter used in each case is indicated above the top panel. Abbreviations: ec, ectoderm; hb, hindbrain; ph, pharynx; y, yolk.

To add further versatility to the assay, we have tested a selection of promoter elements with the I-SceI meganuclease method, using lamprey CNE 3299 as the enhancer. We selected the mouse *β-globin* minimal promoter and three zebrafish minimal promoters from the genes *hsp70*, *klf4* and *krt4* (see Table S1 for sequences), which have previously been shown to display low background activity and high interactivity with a variety of enhancers in zebrafish [37]. No GFP-expressing lamprey embryos were obtained using the *β-globin* or *klf4* promoters with CNE 3299. When tested in conjunction with lamprey CNE 3299, the zebrafish *hsp70*, *krt4* and mouse *c-Fos* promoters all up-regulate GFP expression in a consistent manner in the hindbrain and spinal cord (Figure 3). The consistent GFP expression domains driven by lamprey CNE 3299 with three different minimal promoters confirm the validity of this expression pattern by eliminating the possibility of promoter bias. Each of these three promoters also drives mosaic expression in the ectoderm. The *hsp70* and *krt4* promoters were not tested in the absence of an enhancer, so we cannot definitively pronounce this to be background expression for these two promoters. However, we consider it likely to be background expression as it is in the same domain as that driven by the *c-Fos* promoter control and as described previously for other constructs in lamprey [30].

### Conclusion

We have identified an improved method for lamprey transgenesis. The increased numbers of transient transgenic embryos obtained through the I-SceI meganuclease assay in lamprey makes it an improvement over simple linearised plasmid injection. Given the utility of lamprey as a model organism for investigating early vertebrate evolution and the completion of the lamprey genome assembly, this is both important and timely. This assay will make it possible to probe the lamprey gene regulatory architecture by characterising lamprey enhancers and testing jawed vertebrate regulatory elements for activity in lamprey embryos. Lamprey transgenesis also offers scope for experimental attempts to re-create evolutionary transitions, such as the acquisition of the jaw or paired limbs. In these approaches, jawed vertebrate enhancers could be used to layer novel gene expression domains upon the putatively ancestral embryonic plan of the sea lamprey. The effects of inducing novel genetic cascades in these territories could provide insight into the regulatory changes underlying the evolution of jawed vertebrate characters.

Focusing upon ancient vertebrate CNEs, we have used our lamprey reporter assay to demonstrate conservation and elaboration of CNE function between two distantly related extant vertebrates. Our data from zebrafish and lamprey support the notion that, to a certain degree, ancient conserved enhancer sequences are indicative of core developmental programs that are common to all vertebrates. Nevertheless, these elements also appear to have been susceptible to lineage-specific evolutionary tinkering, with changes in *cis* and *trans* contributing to modification of their regulatory output.

## Supporting Information

Figure S1 **Multiple sequence alignment of CNE 3285 from vertebrate genomes.** Primers used for amplification are highlighted in red.
(PDF)

Figure S2 **Multiple sequence alignment of CNE 3299 from vertebrate genomes.** Primers used for amplification are highlighted in red.
(PDF)

Figure S3 **Comparison of mosaicism from linearised plasmid and I-SceI meganuclease transgenisis approaches.** Examples of stage 24–25 transient transgenic embryos obtained through each approach using the pm3285 enhancer are shown.
(TIFF)

Table S1 Minimal promoters tested in the lamprey reporter assay.
(DOC)
