# Supplementary material for: A Reporter Assay in Lamprey Embryos Reveals Both Functional Conservation and Elaboration of Vertebrate Enhancers
Source: PLoS One. 2014 Jan 9;9(1):e85492. doi: 10.1371/journal.pone.0085492 (PMC3887057; doi:10.1371/journal.pone.0085492)
Supplement: Figure S1 — Multiple sequence alignment of CNE 3285 from vertebrate genomes. Primers used for amplification are highlighted in red. (PDF) [file pone.0085492.s001.pdf]

```
human           ACAGATCTATACAGCACTACCGTTCAGCA-----------AGAAAAAGAGAGTTATATTT
fugu            GTAGATCCATACAGTGCGCCTGTGCGGCGTGGGCGTCGCCAGAGAAGGAGGGTTATATTT
zebrafish       GCAGATCCATACGGCGC-TCGGCTCGGCG-------------ATGAAGAGAGCTATATTT
lamprey         GTGGAGCCTTACAATTGTGCAATTCCGCCCAGACGGCCTCACATTAAGGCGGTGCTTAGG
                   ** *  ***       *    * **              *  * *   *   *

human           TCCCCTTGAGTTAGGACGCCTCATGACAAACGATAATGGATAATCAATAAACTGGGAAAT
fugu            TCCCCTTGAGTTAGGACACCTCGTGACAAATGATAATGGATAATCAATAAAGCGGGAAAT
zebrafish       -CCCCTTGAGTTAGGACACCTCATGACAAATGATAATGGATAATCAATAAAGCGGGAAAT
lamprey         GCAGCCAGAGCAAGAACAGCGCGTGACAAATGATAATGGGGAATCAATACGCGAGAAGAT
                 *  *  ***  ** **  * * ******* ********  ********     * * **

human           ATGAGGCCACAAACTTTATGAAGCCAAGAGACATATTACTTCTGAACAACACTC--ATTT
fugu            ATGAGGCAGCAAACTTTATGAAGCCAGGAGACGAATTACTCCCAAACAACACTATGGTTT
zebrafish       ATGAGGCCGCAAACTTTATGAAGCCGGGAGACGTATTACTCCCCAACAACACTC--GTTT
lamprey         ATCAAGCAGCTGAGCTTATGAAGCTGGGAGACAAATTGCTGGCCTACAACGCTC--ATTT
                ** *  **  *  *  *********   *****  *** **     ***** **    ***

human           CCCTTAACAAAGTCACTACT-AGACTGTGCCTAATAATCTGAAAGAAAATCAAAGGACCT
fugu            CCCTTAACAAAGTCACTACT-CGACTGTGCCTAATAATCTGAAGGAAAATCAAAGGACCC
zebrafish       CCCTTAACAAAGTCACTCCGGCCGCTGCGCCTAATAATCTGAGGGAAAATCAAAGAAGCC
lamprey         CCCTTAACAAAGTCAACATTGCACTTTTTTACACCGTGTTGAAGGAAAATCAAAGAGCCT
                ***************          *      *      ***  ***********   *

human           GCAACCACATCAGCAATATTGGCAACTTATTCCACTTTAATGGCATTTTGATTGATTTTT
fugu            AGAACCACATCAGCAATATTGGCAACTTATTCCACTTTAATGGCATTTTGATTGATTTTT
zebrafish       GCAACCACATCAGCAATATTGGCAACTTATTCCGCTTTAATGGCATTTTGATTGATTTTT
lamprey         T--GCCGGGCTGGTAATATTACCGACTT-TTTCACTCTAATGGCGTTTTGATTGATTTCC
                    **      * * ****  * **** ** * ** ******* *************

human           CTG------CC---------TAATGGTAGTTTTATACTGTACATTTGACGCTGCTTCTGC
fugu            TTT------CCCCCTCTTCCTAATGGTCGTTTTATA-----GATTTGACGCTGCTTCTGC
zebrafish       TCT------CC-----TTCTTAATGGTACTTTTATA-----GATTTGATGCTGGTCCTGC
lamprey         CCCACCCCACCCCCTCACCCTAATGGTAGTTGTATA-----GATTTTGGAATGCTTTCGT
                         **         *******  ** ****      ****     ** *   *

human           AAAATAGTTGTGTGTAATAAACATCCCCGAAGGCAAACA-GTGAACATTAAGGTTCTTGT
fugu            AAAACAGTGGTGTGTAATAAACCTCCCTGAAGGCAAACG-ATAGCCATTAAAGCTCTTGT
zebrafish       AAAACAGCAGTGTGTAATAGAGCTCCCCGAAGGCTAGCA-GCCGCCATTAAAGGCCTTGT
lamprey         AAAATAGCTGTGTGGGGTAAACATCCCAGAAGGCAAAAAAGTCAAAAATATGAGGCATTT
                **** **  *****   ** *  **** ****** *          * **     * * *

human           CTTACTAGGAATCATAATTGAAGCTTGACCAACATTGCCTTTGGCCTTTTAAAAGAAATC
fugu            CTTACTAGCGCTCATAATTGGAGGTTGACCACTACCACATTTTGGCTCCTCACATAACAC
zebrafish       CTTACCAGGACTCGTAATTGGAGGTTGACCACCATCGCATTTCA-CACATCACACAACAC
lamprey         TTGTGAGCAGCTAAAAACTGGAAGCCGGT-AACATAGCTGATTCACCATGAAAAAAATGC
                 *         *   ** ** *    *   *  *   *   *   *     * * **  *
```
