# Supplementary material for: A Reporter Assay in Lamprey Embryos Reveals Both Functional Conservation and Elaboration of Vertebrate Enhancers
Source: PLoS One. 2014 Jan 9;9(1):e85492. doi: 10.1371/journal.pone.0085492 (PMC3887057; doi:10.1371/journal.pone.0085492)
Supplement: Figure S2 — Multiple sequence alignment of CNE 3299 from vertebrate genomes. Primers used for amplification are highlighted in red. (PDF) [file pone.0085492.s002.pdf]

```
human           AAAACAACTTATTTGAAAACTACAAAAA-GTAATTCCATAGAGACTTGCCACTTGACTGA
fugu            ACTACTACACGTAAAAAAAAAAAAAAAAATGTAATTCCGTGGAGACAGCCGGTGTGACTGA
zebrafish       -----------------------------------TATTTATTTGATTGTGTGTGATTGA
lamprey         ----------------------------------CCTTCCGGGCCCGGCCTCCTGACTGA
                                                             *** ***

human           CACCTGTCATCT-CATGATAAATGACTCCATTTTCTGCCTCT-CTTGCCTTCTGCCACT-
fugu            CACCTGTCAGCCGGATGATAAATGACTCCATTTTCTGCCTGT-GCTGCCTTCTGCCACCG
zebrafish       CAGCTGTCGTCCGCATGATGAATGTGTGTGTGTGCTGTGAG-------CGTCTG------
lamprey         TACCTGTCACCG-GATGATAAATGACTCAATTTGCTTTGTAATACCGCCTTCTGTCACT-
                 * *****  *   ***** ****  *   * * **            * ****

human           CCAGCAATGGTCATTTATCATCATCT-CTGTCAGCAATGGAAAGCAGCACTGACAGTGCA
fugu            GCAGCAGTGGCCATTTATCATCATCCGCTGTCAGCCGTGGAAAGCGGCAGTGACAGTGCG
zebrafish       -CAGCTCTGGTCATCCATCATCGTCTGCTGTCACACACATGAAACG-CGGCGACAGCGCC
lamprey         GTGGCCCTGGCCATTCATCATCATCC-CTGTCAGTGGCACAAAGTCGCCCTGACAGGGGA
                   **  *** ***  ****** **  ******        **    *   ***** *

human           AGTCAGCAAACATTTAGCACATGGAACCACGCA-ACACAGGGAAATTATT-ATTGTCAGA
fugu            AGTCAGCAAACATTTAGCACACGTAGCCGCGCT-ACAAGGGGAAATTATT-ATTGTCAGA
zebrafish       AGTCAGCAAACATTTAGCACAGCGAGCCGCACG-GCAGACGGAAATTATT-ATTGTCAGA
lamprey         AGTTGGCAGCCAGTTACTGCACAACACTAGGCCGACACGGGGAAATCGCAGGATGTTAGC
                ***  ***  ** ***   **     *    *   **   ******       *** **

human           ATAATAATG---GTTTAGCATAATTTATTACAGGTC-CACCAAATAAGCAAAGGCTAGA-
fugu            ATAATAATG---GTTTAGCATAATTTATTACAGGTC-CACCAAATAAGCAGGGGCTAGA-
zebrafish       ATAATAATG---CTTTAGCATAATTTATTACAGGTC-CGGCAAATACACTGCGGCTCCG-
lamprey         ATAATAATGGGGGCCGCGAGTGATGCATTATCTGTCGCTGCCAATAAGCCGAGGCCTCAT
                *********        *  * **  ****   *** *  * ****  *   ***

human           -TGTTATTAGACAGATGGATGGGTAGGCTTGGCAGGCCTCCACCGAGACGCAGGCGG---
fugu            -TGTTATTAGACAGATGGATGGGAAGACTTGGCAGACATCCACTGAGACTCGGCCAGGCC
zebrafish       -TGCTATTAGACAGATGGAGAGGCCAGACCGGCAGAAACACACACACA------------
lamprey         CTGCAGCCGGGCAGATGGGCACACCAAAACGGGCAGGCTAGCGGAAGGGG----------
                 **      * *******             *             *
```
