# Supplementary figures and images for: A Reporter Assay in Lamprey Embryos Reveals Both Functional Conservation and Elaboration of Vertebrate Enhancers
Source: PLoS One. 2014 Jan 9;9(1):e85492. doi: 10.1371/journal.pone.0085492 (PMC3887057; doi:10.1371/journal.pone.0085492)

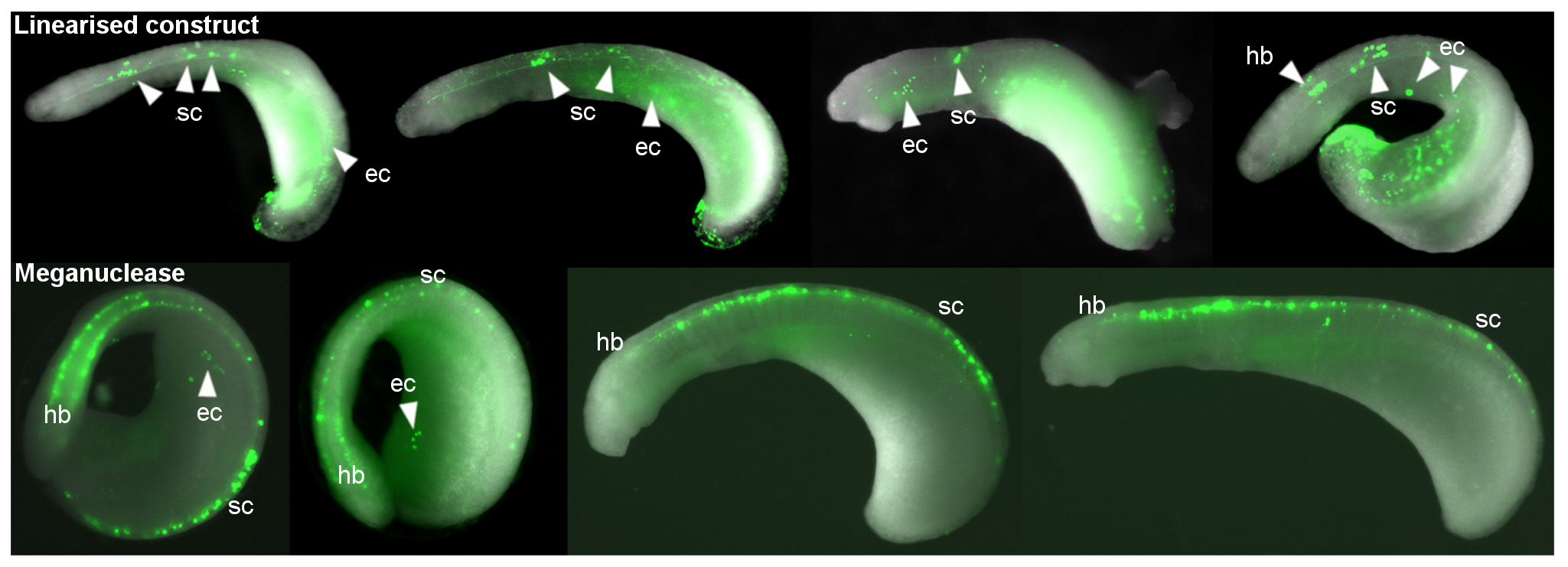

Supplement: Figure S3 — Comparison of mosaicism from linearised plasmid and I-SceI meganuclease transgenisis approaches. Examples of stage 24–25 transient transgenic embryos obtained through each approach using the pm3285 enhancer are shown. (TIFF) [file pone.0085492.s003.tiff]
