# Supplementary material for: A Reporter Assay in Lamprey Embryos Reveals Both Functional Conservation and Elaboration of Vertebrate Enhancers
Source: PLoS One. 2014 Jan 9;9(1):e85492. doi: 10.1371/journal.pone.0085492 (PMC3887057; doi:10.1371/journal.pone.0085492)
Supplement: Table S1 — Minimal promoters tested in the lamprey reporter assay. (DOC) [file pone.0085492.s004.doc]

| Promoter name | Species of origin | Sequence |
| --- | --- | --- |
| cfos | mouse | CCAGTGACGTAGGAAGTCCATCCATTCACAGCGCTTCTATAAAGGCGCCAGCTGAGGCGCCTACTACTCCAACCGCGACTGCAGCGAGCAACTGAGAAGACTGGATAGAGCCGGCGGTTCCGCGAACGAGCAGTGACCGCGCTCCCACCCAGCTCTGCTCTGCAGCTCCCACCAGTGTCTACCCCTGGACCCCTTGCCGGGCTTTCCCCAAACTTCGAGGATCC |
| β-globin | mouse | CCCGGGCTGGGCATAAAAGTCAGGGCAGAGCCATCTATTGCTTACATTTGCTT |
| hsp70 | zebrafish | TTGATTGGTCGAACATGCTGGCGCGTTCGAGAAACTTCAGCGGCGGCTTGCTGGAAAATATAAAAGAGCAGCTGTTTCCAGTCCAGTACTCCAGCATAGACTTCGCGATAGAACTGTATACAGCGGAAAGCGAGACAGCGAGCGGACTG |
| krt4 | zebrafish | CAAGTGTGTGTGTGAGAGCAGTCAGCTCCACCCCCTCAAGAGTGTGTATAAAATTGGTCAGCCAGCTGCTGAGAGACACGCAGAGGGACTTTGACTCTCCTTTGTGAGCAACCTCCTCCACTCACTCCTCTCTCAGAGAGCACTCTCGTACCTCCTTCTCAG |
| klf4 | zebrafish | ACTACATCCCAAGCGTCATGAGAACACGCCCACTATGCATTCAGACCCTCCTTATAATGGGACAGGTAGACATCCAGCCAAACATATTAGAGACGCATTGTCTGCCTCGGACTGAATGAGAGTAAACACCT |
